# Editorial: Endocrine abnormalities and renal complications

**DOI:** 10.3389/fendo.2023.1274669

**Published:** 2023-08-21

**Authors:** Anil Kumar Pasupulati, Sreenivasulu Kilari, Manisha Sahay

**Affiliations:** ^1^ Department of Biochemistry, University of Hyderabad, Hyderabad, India; ^2^ Vascular and Interventional Radiology Translational Laboratory, Department of Radiology, Mayo Clinic, Rochester, MN, United States; ^3^ Department of Nephrology, Osmania Medical College and General Hospital, Hyderabad, India

**Keywords:** kidney, hormones, chronic kidney disease, acute kidney injury, biomarkers, end-stage kidney disease

The principal function of the kidneys is to keep the blood clean and chemically balanced by filtering out waste products of metabolism and maintaining water, electrolyte, and acid-base balance. Kidneys help retain glucose, amino acids, vitamins, hormones, albumin, antibodies and other vital components in the blood while eliminating urea and creatinine. The kidneys’ ability to elicit selective filtration and tight regulation of the composition of urine is accomplished by the glomerulus and tubular region of the nephron, the functional unit of the kidney. The severity of impairment of renal function is characterized by decreased glomerular filtration rate (GFR) and albuminuria, which are markers for kidney excretory function and glomerular barrier dysfunction, respectively. Further, impairment of renal function is characterized by elevated serum creatinine (SCr), cystatin C or blood urea nitrogen (BUN). Several endocrine factors secreted by kidneys regulate various physiological functions. Kidneys synthesize renin to maintain arterial blood pressure, whereas excess renin secreted by the injured kidney contributes to hypertension. Erythropoietin secreted by the kidney stimulates the production of RBC by the bone marrow, and in conditions of reduced kidney function, insufficient erythropoietin results in anemia. Notably, decreased kidney function is often presented with anemia and is associated with a reduced quality of life and increased morbidity (1). 25-hydroxyvitaminD(3)-1alpha-hydroxylase (*CYP27B1*) produced by proximal tubular cells of the kidney catalyzes the synthesis of calcitriol, the most active form of vitamin D (1,25-dihydroxyvitamin D), which plays an integral role in calcium homeostasis. Because of their multiple roles, kidneys are considered life-sustaining organs. The articles on this topic provide a comprehensive understanding of kidney function in various endocrine abnormalities, discuss the intricate role of intrarenal and systemic endocrine factors on kidney function, and investigate strategies for early and specific diagnosis of renal complications.

Kidney diseases are a modern-day epidemic, a direct cause of morbidity and mortality and a significant risk factor for cardiovascular disease (CVD) (2). Acute kidney injury (AKI) and chronic kidney disease (CKD) are two kinds of kidney complications. AKI is presented with an abrupt decline in GFR (hrs to days) and is diagnosed with any one of the following: an increase in SCr by 0.3 mg/dl within 48hrs or an increase in SCr to 1.5 times baseline within the prior seven days, or urine volume less than 0.5 ml/kg/hr over 6 hrs (3). Some common causes of AKI include exposure to nephrotoxins, ischemia, pre-renal diseases such as hypotension, hypovolemic states, urinary tract obstruction, and viral infection such as COVID-19. AKI is frequent among hospitalized patients in the intensive care unit setting. Though AKI is asymptomatic in many patients, it is associated with thirst, dehydration, low or no urine volume, hematuria, edema, and shortness of breath. On the contrary, CKD is a long-term condition with a gradual loss of kidney function, as evidenced by a five-stage (1-5) progressive decline in GFR (4). As per Kidney Disease Improving Global Outcomes (KDIGO) guidelines, an individual is considered to have CKD if abnormalities of kidney structure or function persist for more than three months (5). End-stage kidney disease (ESKD) is the worst CKD stage and a significant public health concern. CKD is irreversible and eventually requires permanent dialysis or kidney transplant. Mortality increases with decreasing GFR and increasing albuminuria. CKD is associated with symptoms such as anemia, hypertension, edema, lethargy, persistent headaches, lower back pain, and growth delay in children.

Several factors can contribute to the pathogenesis of CKD. Hypertension, diabetic kidney disease (DKD), obesity, and aging are the most common causes, whereas factors such as HIV, exposure to toxins, and heavy metals contribute to the burden of CKD. In some areas of developing countries where CKD is endemic, precise causal factors remain to be established. These scenarios contribute to different factors that manifest in long-term glomerular and tubular damage and ultimately manifest in ESKD. In diabetes, elevated pituitary growth hormone (GH) circulatory levels are implicated in impaired renal function. Receptors for GH and its mediator IGF-1 are abundantly expressed in glomerular and tubular cells. GH can act directly on the kidneys or via circulating or paracrine-synthesized IGF-1. The GH/IGF-1 system regulates glomerular hemodynamics, tubular sodium and water, phosphate, calcium handling, and renal gluconeogenesis. The GH/IGF-1 system governs klotho synthesis, a coreceptor of the phosphaturic hormone fibroblast growth factor 23 (FGF-23) in the renal tubule. Although recombinant GH is widely used in treating short stature in children, including those with CKD, studies from experimental animals and acromegalic patients demonstrate that GH excess can have harmful effects on the kidney, including glomerular hyperfiltration, renal hypertrophy, and glomerulosclerosis (6). In addition, elevated GH in patients with poorly controlled type 1 diabetes mellitus was thought to induce podocyte injury and contribute to diabetic nephropathy. The direct action of GH mediates these adverse effects predominantly in the glomerulus, and by contrast, IGF-1 excess results in tubular hypertrophy only. The pleiotropic effects of GH on podocytes include expression of pro-sclerotic TGF-β, pro-inflammatory TNF-α, activation of epithelial-mesenchymal activation, and cell death by mitotic catastrophe (7–10).

Although the thyroid hormones (TH) contribute to the development of the kidney, both hypo- and hyperthyroidisms affect GFR, renal blood flow, tubular function, and electrolyte balance. Hypothyroidism is associated with a reversible increase in serum creatinine, reduced GFR and renal plasma flow. If hypothyroidism can be corrected with levothyroxine therapy, some individuals’ blood creatinine levels can return to normal (Zhang et al.). In comparison, hyperthyroidism is associated with increased GFR and renal plasma flow. In patients with CKD, low T3 levels are independent predictors for all-cause mortality in euthyroid patients with ESKD. TH-TH receptor (TH-TR) axis alterations are critically involved in the pathogenesis of DKD. Despite low T3 levels, patients of DKD are presented with reexpression of fetal isoform TRα1 in podocytes and are concomitant with maladaptive cell-cycle induction/arrest (11). Interestingly, T3 treatment reduced TRα1 expression and mitigated podocytes’ maladaptive response (11).

Abnormalities in the synthesis of estrogen and progesterone are common in women with CKD (12). Abnormal menstrual cycles with amenorrhea, anovulation, and early menopause are often observed in women with CKD. Diagnosis and management of menopausal symptoms and postmenopausal osteoporosis in CKD remain challenging. Testosterone deficiency and testicular dysfunction are frequent among men with CKD. Testosterone levels decline as CKD progresses with further reductions in GFR. Combined evaluation of the GFR and circulating testosterone improves mortality risk. Both men and women with CKD also suffer from decreased fertility. The relationship between sex hormones and kidney stone formation is a topic of debate because urolithiasis was observed more in men compared with women. It was largely believed that testosterone is the main reason for urolithiasis that observed predominantly among men. Huang et al. reported that serum testosterone levels were inversely associated with the prevalence of kidney stones in men over 40. The association of endogenous sex hormones and sex hormone-binding globulin (SHBG) with CKD was investigated by Lau et al. Among men, no associations were observed between androgens, eGFR, and CKD. In women, a higher T/DHT (Testosterone/dihydrotestosterone) ratio was associated with higher CKD prevalence and that higher circulating levels of free DHT were associated with a lower incidence of CKD.

The complex interplay among parathyroid hormone (PTH), calcitriol, and FGF-23 help regulate the normal serum calcium (Ca) and phosphorous (P) levels. Kidneys play an instrumental role in maintaining serum Ca and P levels by regulating these three hormones. CKD-related mineral bone disorder (MBD) represents a complex disease with elevated PTH and FGF-23, reduced levels of calcitriol and klotho (13). This adaptive endocrine response maintains serum levels of Ca and P in the normal range until the advanced stages of CKD, where hypocalcemia, hyperphosphatemia, renal osteodystrophy, and vascular calcification are evident. Lee et al. demonstrated deficiency of serum 25-hydroxy vitamin D levels was significantly associated with only severe CKD stage (4&5) among the Korean cohort. Vitamin D receptor agonists, nutritional vitamin D, and calcimimetic agents to reduce parathyroid hormone are prescribed to treat CKD-MBD and to influence the survival rate in patients with ESKD. Notably, elevated serum PTH levels were significantly associated with an increased risk of peritonitis in the Chinese cohort undergoing continuous ambulatory peritoneal dialysis (Zhao et al.). Further, Kee et al. evaluated the influence of residual kidney function in patients undergoing prevalent hemodialysis on the detrimental effect of serum FGF-23 levels in CVD development.

Dyslipidemia and abnormal lipid metabolism contribute to kidney cell injury, increasing the risk of CKD in obese individuals (14). Several obesity indices are available, including body mass index, waist circumference, and visceral adiposity index (VAI). Lin et al. report that higher CVAI is associated with an increased risk of renal damage (as assessed in terms of eGFR and proteinuria) in patients with hypertension and abnormal glucose metabolism. Furthermore, CVAI strongly predicts renal damage incidence compared with the above mentioned indices. Therefore, a simple assessment of visceral adiposity by calculating CVAI may be helpful for the early identification of high-risk individuals and for adopting strict BP and glucose management, thereby reducing the risk of renal damage. Serum apolipoprotein B (ApoB) levels had the strongest correlation with CKD among all lipid variables, and accumulation of ApoB levels might precede the occurrence of CKD (Xu et al.).

Adiponectin (A) and leptin (L) are two hormonally active molecules secreted by adipose tissue and are critical mediators of cardiometabolic risk in obesity (15). In healthy individuals, adiponectin elicits anti-inflammatory effects and is cardioprotective, whereas leptin is associated with obesity-related cardiovascular complications and pro-inflammatory activity. Nevertheless, paradoxically, in CKD patients, A, L, and the ratio of L/A are increased, independently of traditional CKD risk factors. In the settings of CKD, elevated adiponectin levels are associated with decreased bone mineral density, anemia, and hypertrophy of the left ventricle. At the same time, elevated leptin levels are associated with endothelial dysfunction and aortic stiffness; adiponectin and leptin contribute to a higher risk of CVD in CKD. Graňák et al. revealed A/L(< 0.5) as a predictor of acute rejection in the early post-transplant period after kidney transplantation.

Considering the magnitude of the global burden of kidney disease and the seriousness of morbidity and mortality of ESKD, we solicit biomarkers that help early diagnose the individuals at high risk of renal complications with endocrine abnormalities, in addition to intervention strategies. Cao et al. performed two independent cross-sectional studies to explore whether plasma levels of urea cycle-associated amino acids with risk of DKD. According to this study, plasma citrulline levels were significantly associated with the risk of DKD in type II diabetes in the Chinese population. The protein concentration of urinary extracellular vesicles (UEV) in diabetic individuals is higher than in healthy controls before and after adjusting the urinary creatinine (UCr) (Gu et al.). The ratio of uEV-to-UCr may better indicate the progression of diabetic renal complications over the urine protein–Cr ratio or albumin-Cr ratio. Since albuminuria is associated with high glycemic variability in type II diabetic patients, avoiding fluctuations of blood glucose levels using flash monitoring methods could help better manage renal health among the diabetic population in the Indian cohort (Nathiya et al.).

## Author contributions

AP: Writing – original draft, Writing – review & editing. SK: Writing – review & editing. MS: Writing – review & editing.
